# Detecting Precontact Anthropogenic Microtopographic Features in a Forested Landscape with Lidar: A Case Study from the Upper Great Lakes Region, AD 1000-1600

**DOI:** 10.1371/journal.pone.0162062

**Published:** 2016-09-01

**Authors:** Meghan C. L. Howey, Franklin B. Sullivan, Jason Tallant, Robert Vande Kopple, Michael W. Palace

**Affiliations:** 1 Department of Anthropology, University of New Hampshire, Durham, NH, United States of America; 2 Earth Systems Research Center, Institute for the Study of Earth, Oceans, and Space, University of New Hampshire, Durham, NH, United States of America; 3 University of Michigan Biological Station, Pellston, MI, United States of America; 4 Department of Earth Science, University of New Hampshire, Durham, NH, United States of America; Max Planck Institute for the Science of Human History, GERMANY

## Abstract

Forested settings present challenges for understanding the full extent of past human landscape modifications. Field-based archaeological reconnaissance in forests is low-efficiency and most remote sensing techniques are of limited utility, and together, this means many past sites and features in forests are unknown. Archaeologists have increasingly used light detection and ranging (lidar), a remote sensing tool that uses pulses of light to measure reflecting surfaces at high spatial resolution, to address these limitations. Archaeology studies using lidar have made significant progress identifying permanent structures built by large-scale complex agriculturalist societies. Largely unaccounted for, however, are numerous small and more practical modifications of landscapes by smaller-scale societies. Here we show these may also be detectable with lidar by identifying remnants of food storage pits (cache pits) created by mobile hunter-gatherers in the upper Great Lakes during Late Precontact (ca. AD 1000–1600) that now only exist as subtle microtopographic features. Years of intensive field survey identified 69 cache pit groups between two inland lakes in northern Michigan, almost all of which were located within ~500 m of a lakeshore. Applying a novel series of image processing techniques and statistical analyses to a high spatial resolution DTM we created from commercial-grade lidar, our detection routine identified 139 high potential cache pit clusters. These included most of the previously known clusters as well as several unknown clusters located >1500 m from either lakeshore, much further from lakeshores than all previously identified cultural sites. Food storage is understood to have emerged regionally as a risk-buffering strategy after AD 1000 but our results indicate the current record of hunter-gatherer cache pit food storage is markedly incomplete and this practice and its associated impact on the landscape may be greater than anticipated. Our study also demonstrates the potential of harnessing commercial-grade lidar for other fine-grained archaeology applications.

## Introduction

**Fig 1:**
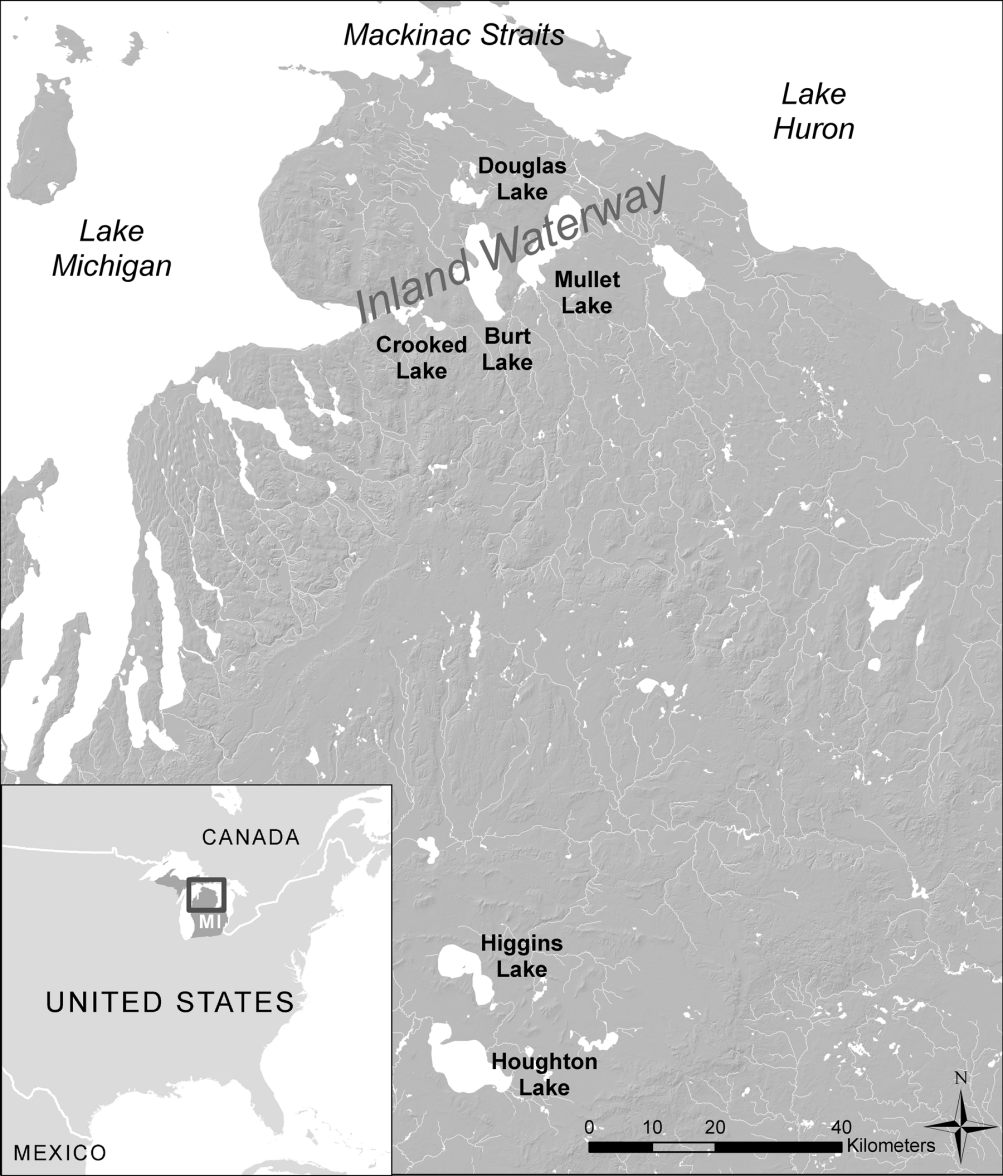
Location of Douglas and Burt Lakes study area in the upper Great Lakes region (Base data from Data and Maps for ArcGIS (ESRI) and the National Elevation Dataset (NED)).

Forested landscapes pose significant challenges for understanding the full extent of past human activities and modifications of the landscape. Field-based archaeological surveys in forests are often arduous and time-intensive, resulting in restricted spatial coverage. Most available remote sensing survey techniques are also of limited utility for finding archaeological features under forest canopies (e.g., airborne and satellite platform optical sensors). As a result, many archaeological sites and formations in forested settings are simply unknown [1–3]. Lidar (LIght Detection And Ranging) represents the potential for a paradigm shift in our approach to archaeology, and here, we show how it may be specifically so for studying the microtopographic impacts of mobile hunter-gatherers in the forested landscapes of the upper Great Lakes (Fig 1).

Lidar, a remote sensing tool that uses pulses of light to measure the elevation of reflecting surfaces, has increasingly served to further archaeological research. It has been suggested that its expanded application could transform the discipline in ways comparable to the advent of radiocarbon dating [4]. This remote sensing technique stands to aid in our understanding of human modifications of landscapes across broad scales due to its high accuracy irrespective of overlying vegetation and because it can be tuned to varying landscape types and applications [4–8]. Some archaeological research has already capitalized on the ability of lidar to penetrate vegetation cover with strong results [1–4, 9–12]. These studies have focused largely on complex past societies; that is, societies that were relatively high in population density, hierarchical, agriculturalist, sedentary, and which built a variety of permanent structures. It is reasonable that in archaeological studies using lidar there has been such focus on complex societies because these communities left a definitive material signature on the landscape that is distinguishable from natural topographic features even in a forested setting. Certainly, the results of such research have borne this out through the finding of distributions of monuments, buildings, terracing, and other anthropogenic modifications more extensive than anticipated, especially in dense tropical forests [4,10].

Not all past societies were complex, however, and over the course of human history a diverse range of relatively low-density, egalitarian, mobile hunter-gatherer societies existed across the globe. While early research conceived of these as simple societies on a low rung of a progressive social evolutionary ladder, more contemporary research recognizes that mobile hunter-gatherers were dynamic and had, if not complex, still complicated cultural systems [13,14]. Even as such recognition grows, remnants of early conservative interpretations still influence archaeological research on mobile hunter-gatherer societies, especially in terms of appreciating the scope, scale, and extent of anthropogenic shaping these societies were capable of doing to the natural world [15].

Recent studies have presented cases where mobile hunter-gatherers overtly modified the landscape, like through the building of mounds and earthworks [16], but more practical modifications of the natural world were also routine and these can be reflected in microtopography, e.g. of specific interest here, subterranean pits for food storage (cache pits) in the upper Great Lakes. Although cache pits did not involve macroscale alterations of the earth, the persistent construction of these mundane features was nevertheless a critical way past mobile hunter-gatherers shaped the landscapes they inhabited in order to mitigate against episodes of scarcity, ensure survival, and increase social well-being [17–23]. The significance of these anthropogenic microtopographic modifications in past socioecological processes remains to be fully fleshed out.

**Fig 2:**
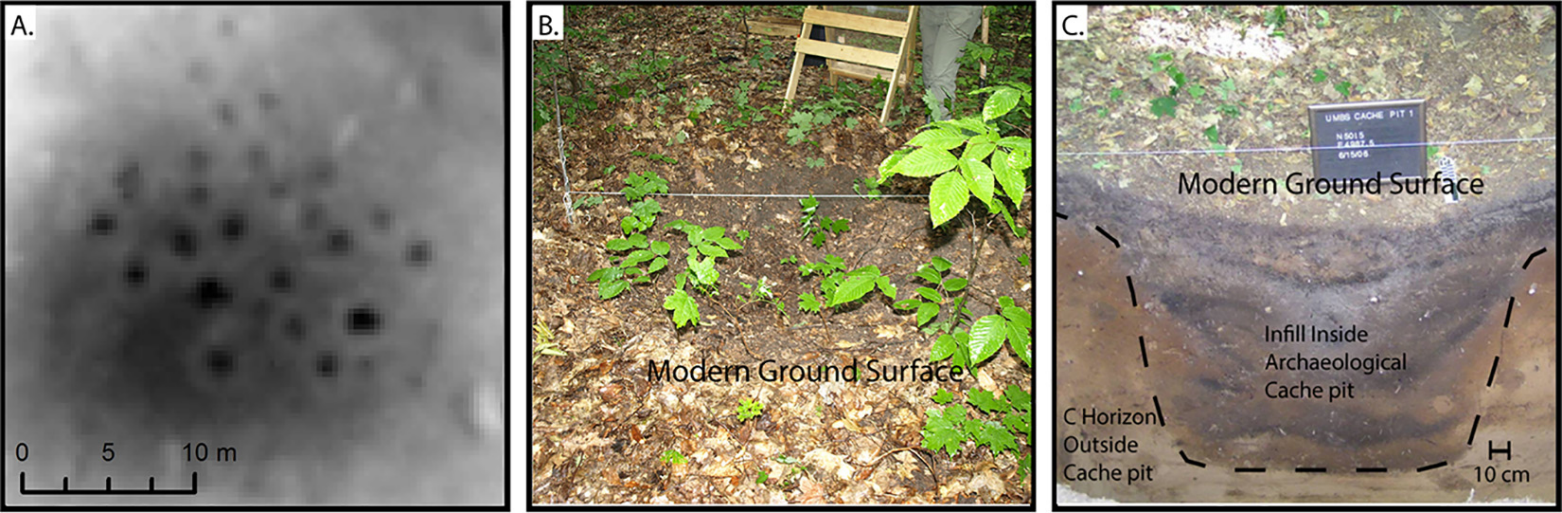
**A. Image on high resolution digital terrain model (DTM) of one previously field identified cache pit cluster. B. A cache pit with leaf litter cleared for cross-section excavations showing current appearance of these as archaeological features. C. Cross-section excavation showing the full profile of an archaeological cache pit.**

In order to better grasp their importance, we would benefit from a more complete record of cache pit features across local and regional landscapes. Ethnohistoric accounts indicate cache pits in active use were subterranean u-shaped features, ranging from 1 to 2 meters wide and deep, lined with birch bark, filled with multiple foodstuffs in containers with grass-type fill in between, and capped with mounded dirt [24]. Cache pits predominantly survive as archaeological features in the upper Great Lakes in forested areas subject to less post-EuroAmerican disturbance and they do so as subtle, low-relief circular depressions with diameters ranging from 1 to 2 meters and current surface depths between 0.3 to 0.6 meters (Fig 2). A variety of factors make using standard field-based archaeological reconnaissance techniques to expand and improve the cache pit record a burdensome task. Communities dispersed food storage away from where they lived for use in annual mobility rounds and this means cache pits are not readily encountered during archaeological research on past habitation sites. Cache pit features are easily obscured by leaf litter and groundcover, making it hard to find them until standing almost right on top of them (Fig 2). Moreover, cache pits share morphological similarities with treethrow, or pit-and-mound topography, a type of microtopography very common in eastern deciduous natural-forested ecosystems [25, 26]. Two key factors make it possible to distinguish cache pit anthropogenic microtopographic features from these much more abundant natural ones. One, cache pits have a more uniform circular shape and lack the mounding up of treethrow pits and two, cache pits almost always occur in regularized clusters, typically with 10 to 25 pit depressions (but sometimes hundreds). While these factors make it possible to distinguish cache pit topographic features from pit-and-mound ones, it still requires a trained eye to do so reliably in archaeological field survey.

## Materials and Methods

The State of Michigan recently launched a lidar collection program, Michigan Statewide Authoritative Imagery & Lidar (MiSAIL), which provided an opportunity to test the utility of lidar to expand insight on the extent of these fine-scale microtopographic features in one inland lake landscape within the upper Great Lakes, Douglas and Burt Lake (Fig 1). Our field-based archaeological research program began in 2007 here, stationed out of the University of Michigan Biological Station (UMBS). The only previous archaeological research in this area focused on finding habitation sites and did not identify any cache pits [27, 28]. Cache pits were first documented on Burt Lake in 1987 during a UMBS research program on pre-EuroAmerican landcover [29]. Our team’s research was the first follow-up work done. The most efficient time to find cache pits using traditional walk-over archaeological survey methods is in the late spring because the forest groundcover has not come up and snow laying in surface depressions melts slower than on the ground which makes cache pits more obvious. UMBS Resident Biologist Robert VandeKopple has led a team of citizen scientists in spring survey (results confirmed during summer). Survey methodology involves walking with GPS units in 50 meter transects along shore and nearshore landforms. To date, 69 clusters of cache pits have been found this way. We conducted expanded field examination of a sample of these pits through cross-section excavations (Fig 2; see [22] for details on these excavations; field work conducted on UMBS property, with permission from UMBS).

The results of this work indicate these cache pits date to the Late Precontact period (ca. AD 1000–1600) which is when cache pits became a key scarcity mitigation strategy across the upper Great Lakes [22]. If we had not specifically looked for them, cache pits would erroneously be considered an insignificant part of the area’s archaeological record and we would not understand that physical food storage formed a major aspect of past day-to-day life here. While the record of cache pits we have established with field-based survey is significant, it still has been slow to produce results for a relatively small region and large areas around these lakes (and across the upper Great Lakes region as a whole) remain unexamined for these features.

### Lidar collection and digital terrain model

Discrete return lidar data were collected during leaf-off conditions in the spring of 2015 as part of the Michigan Statewide Authoritative Imagery & Lidar (MiSAIL) program. The Sanborn Map Company was tasked by the State of Michigan to deliver high point density returns with Nominal Point Spacing of 0.71 m. Data were collected using a Leica ALS80 sensor flown on board an aircraft at an altitude of approximately 1200 m with a maximum scan angle of 32 degrees. Lidar acquisition was in compliance with Quality Level 2 of the United States Geologic Survey (USGS) National Geospatial Program (NGP) Base Lidar Specifications [30]; horizontal accuracy of non-vegetation returns were better than 1 m and vertical accuracy of non-vegetation returns was 0.029 m. The lidar output standard for MiSAIL is a one meter Digital Terrain Model (DTM) which would leave relevant microtopograpic features unidentifiable. We found that, in fact, the resulting lidar data were suitable for generation of 1 foot contours (0.305 meter) (which we acquired as an LAS Dataset which is the industry-standard binary format for storing airborne lidar data).

Classified point clouds were processed in ArcGIS Desktop 10.3.1 (ESRI 2015. ArcGIS Desktop: Release 10.3.1. Redlands, CA: Environmental Systems Research Institute) using the LAS Dataset and Conversion toolsets. A digital terrain model (DTM) was derived by creating a new LAS Dataset layer consisting of a filtered subset of points classified as American Society for Photogrammetry and Remote Sensing (ASPRS) Class 2 (Ground) [31]. These bare earth points were exported to a raster file in TIFF format (.tif), with an output cell resolution set to 1 foot (0.305 meter), with natural neighbor triangulation interpolation and no point thinning.

### Pit delineation

We developed an automated routine to delineate candidate caches in DTMs via a two-step process (S1 Fig). First we normalized the DTM by performing a 5x5 moving window median convolution and subtracted the resulting image from the DTM. We then performed a convolution on the resulting median-removed DTM using a 5x5 kernel (S2 Fig) which emphasized differences between the center pixels and their neighbors, accentuating low-lying areas. The resulting image (DTMmod) ranged from highly negative values to highly positive values, where zero indicates little elevation change between the center pixels and the neighborhood, positive values indicate the center pixels are higher than the neighborhood, and negative values are indicative of lower elevation terrain relative to the neighborhood (the middle panel of S1 Fig offers an example of DTMmod). Anomalously low values are locally low-lying areas, which represent potential cache pits. Using a threshold of -50, we generated a binary image of low-lying (pit) and non-pit pixels. Because cache pits are typically regularly shaped and larger than the pixel size, we clumped the binary image using all neighboring and diagonal pixels to calculate the number of neighboring pixels classified as pits for each pit classified pixel. We removed pit pixels that had fewer than four neighboring pixels classified as pits, then polygonised the final image to a shapefile to represent all candidate cache pits.

For each candidate cache, we calculated a series of parameters to describe its shape and size, including perimeter-area ratio (L/S), corrected perimeter-area (CPA) and related circumscribing circle (RCC), the radius of the circumscribing circle (radius), and the perimeter and area of the polygon [32]. We also calculated zonal statistics for each polygon from DTMmod and a map of the terrain ruggedness index (TRI), which we derived from the DTM. Because caches and pits resulting from treefalls are visually similar to each other, the resulting images and shapefile were examined to manually identify caches that were delineated by the routine which had been observed during our team’s archaeological field survey. We used the manually identified caches and non-caches to characterize the constraints on the shape, size, and terrain characteristics of caches as compared to non-caches with a logistic regression in R (v 3.0.1, R Foundation for Statistical Computing, Vienna, Austria, http://www.R-project.org/). Using the probability calculated from the results of the logistics regression and the area of each cache, we performed a pseudo accuracy assessment on the manually interpreted sample of caches. Accuracy was calculated as:

$$A_{ap}=\frac{R}{R+O+C}$$

where A is the accuracy at the assigned threshold level for area, a, and probability, p; R is the number of confirmed caches retained; O is the number of confirmed caches omitted; and C is the number of non-caches committed. We reduced the candidate pits to a set of likely cache pits using the thresholds determined from the accuracy assessment (S1 and S2 Tables).

### Cluster delineation

To better understand the distribution and clustering of individual cache pits, we calculated the kernel density using the Kernel Density tool in the Spatial Analyst Toolbox in ArcGIS 10.1, using a 25 foot (7.62 meters) search radius weighted by cache pit area with an output cell size of 1 foot (0.305 meters). We used a threshold of 0.06 to identify distinct areas of relatively high density, which we considered as possible discrete cache pit clusters. We selected a subset of the identified candidate clusters for a logistic regression, using the locations of the cache clusters previously identified in our team’s archaeological field survey to confirm delineated clusters as cache pits and manual interpretation to identify false positives. Logistic regression was performed using a set of variables describing the shape, size, and terrain variability of each candidate cluster and the cache pits within. Using the results of the logistic regression, we performed a pseudo accuracy assessment to reduce the candidate clusters to a set of likely cache pits.

One of the surest ways to rot food in a Great Lakes subterranean storage pit is saturation by water. Previous work used a Compound Topographic Index (CTI), which is an index of water accumulation in soils, and found locating cache pits where they had reduced likelihoods of becoming saturated by water was a critical factor in storage success [21]. Following the results of [21], we created lidar-derived CTI maps to determine if the CTI within delineated cache clusters was significantly different from random, which would evaluate locational preference of caches related to dryness or wetness of soil.

We resampled our 1-foot (0.305 meter) DTM to 10 m, 30 m, and 90 m, and calculated CTI at each spatial resolution. We then compared CTI values of pixels that overlapped with cache clusters to CTI values of pixels coinciding with 350 randomly generated locations throughout the extent of the DTM using two nonparametric tests: the Kolmogorov-Smirnov, which allows for testing of differences in distributions of two samples of continuous observations, and Mood’s median test, which allows for testing of the equality of medians from two or more populations. Lower CTI scores indicate improved drainage. In every iteration of the comparison of CTI values of pixels containing cache pits and pixels underlying randomly selected coordinate pairs, cache pits were statistically significantly located on lower CTI scores (S3 Table). Using these results, we generated a mask to remove candidate clusters which were occurring in areas with standing water and soil saturation, factors posing functional limits for the presence of cache pit storage. All statistical analyses were performed in R v 3.0.1.

## Results

We found a significant relationship between pit parameters and the pit/non-pit classification using radius, area, perimeter, L/S, TRI, and DTMmod (p<0.001 for all variables and the intercept). By performing a pseudo accuracy assessment, we found that high accuracy and high percentage of true caches retained was by using probability and area thresholds of 0.58 and 13 ft^2^, respectively (S1 Table). Applying these thresholds, we filtered the number of candidate caches from 2,532,189 to 177,195 within the DTM extent.

From the kernel density analysis, we identified 13,686 candidate clusters of cache pits. The logistic regression resulted in a significant relationship with variables describing the mean perimeter of caches within the delineated cluster (perimeter), the mean value of DTMmod within caches (cache DTMmod), the mean value of DTMmod within the delineated cluster (cluster DTMmod), and the mean TRI within the delineated cluster (cluster TRI) (p < .05 for intercept and cluster DTMmod, p < .01 for perimeter and cache DTMmod, p < .001 for cluster TRI). Following the pseudo accuracy assessment (S2 Table), we filtered the candidate cache clusters from 13,686 to 1593 using a probability threshold of 0.51 (the right panel in S1 Fig offers an example of what pits were filtered from the candidate cache data set in this process).

Using the Kolmogorov-Smirnov test, we determined that there is a significant difference (p<0.001) between lidar-derived CTI for cache clusters at all three scales (10m, 30m, 90m), with increasing disparity between the distributions as pixel size increases (S3 Table). In addition, Mood’s median test shows significant differences between the median CTI for cache pixels and random pixels at each of the scales (p<0.001), with the CTI range decreasing as pixel size increases (S3 Table). Because the scale of the largest clusters of cache pits occur on scales most similar to the 90 m pixels, we used this to generate a CTI mask for cache pit clusters. With the CTI mask, we further reduced the candidate cache clusters to 543.

**Fig 3:**
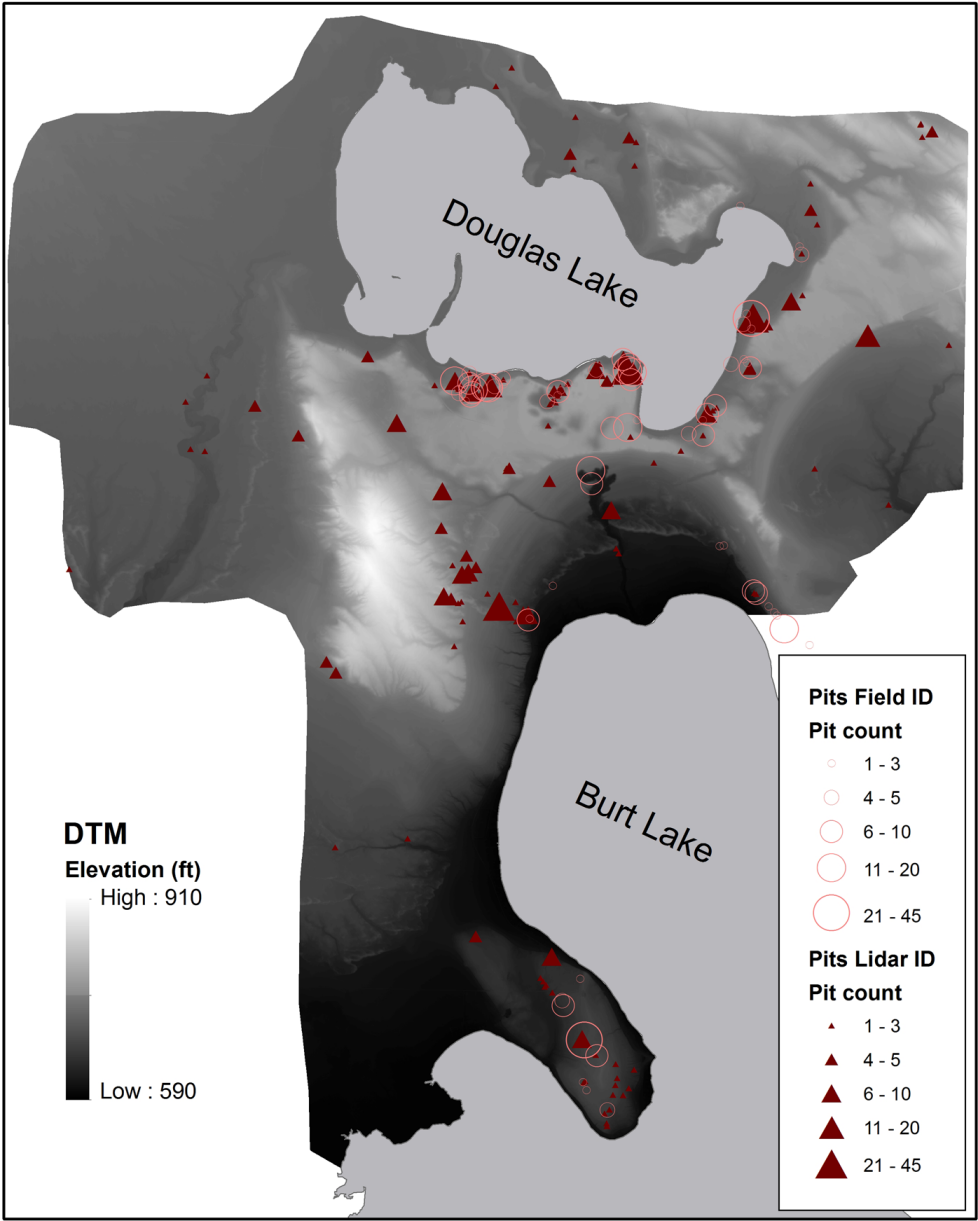
Field survey identified cache pit clusters shown together with lidar identified cache pit clusters on the DTM extent around Douglas and Burt Lake (note, scale bar omitted to reduce locational detail that could be used in inappropriate access to sensitive archaeological features).

We visually identified 139 of these 543 candidate clusters as having a high potential to be anthropogenic cache pit features (refer to Fig 2 for an up-close image of the DTM visual appearance of one cache pit cluster known from field survey; S3 Fig offers an example of what clusters were manually filtered out based on visual assessment). Within these 139, our field surveyed cache pit clusters are closely tracked, with either the original GPS point marks collected during survey directly included in our lidar derived cluster polygon or occurring in close proximity to them. This indicates successful identification of zones of cache pit placement in the landscape (five pits from previous survey were not included on the DTM coverage available to us) (Fig 3). We have ground-truthed eight previously unknown candidate cache pit clusters from the 139 found in our lidar routine and all were field verified as cache pits. Interestingly, many newly identified clusters are quite distant from the inland lakes compared to almost all previously discovered cultural activity sites (Fig 3). We would never have examined such areas removed from the lakes without the aid of lidar and image processing routines.

## Discussion

Our study indicates that cache pits, relatively subtle archaeological features produced by mobile hunter-gatherers, can be identified through advanced analyses of commercial-grade lidar data. Very few archaeological research programs can acquire research-grade lidar, but access to commercial-grade lidar is increasing globally as state-sponsored programs collect lidar data for other applications. Our case not only demonstrates the potential of lidar for fine-grained archaeology applications, but also corroborates the notion that DTM generation should be parameterized for individual use-cases. Indeed, without end-user involvement in the processing of point-cloud data, subtle archaeological features like cache pits can be filtered out of output DTMs entirely [5], as they are smaller than the spatial resolution of the produced map.

As lidar becomes available across the upper Great Lakes, our work demonstrates a capability to examine vast geographic swaths for microtopographical archaeological features, such as cache pits. Field survey can then be focused on areas with higher probability of such features, rather than time consuming walk-over survey in forests. Given that we have found several new cache pit clusters in a landscape that has seen some of the most intensive field surveys ever done for cache pits, the possibility of finding numerous previously unknown cache pit clusters across the upper Great Lakes region is high. As more past anthropogenic microtopographic features are found, it raises the question of the impacts such modifications may carry for ecological processes, which may warrant consideration not just by archaeological research programs. What this study makes clear is that, at best, we have a markedly incomplete record of the ways mobile hunter-gatherers of the upper Great Lakes regularly modified the natural world to ensure their own well-being. As we utilize tools like lidar to expand this record, our interpretations of past social and economic developments among these relatively low-density, mobile societies will have to change and with this, the remnant influence of early conceptualizations of such societies as simple will be further dissolved.

## Supporting Information

S1 Fig Lidar and modified images for an approximately 5000 ft^2^ (483 m^2^) area on the east side of Douglas Lake. The lidar DTM (left) shows slight depressions, accentuated in DTMmod (middle), which was used to generate a binary image of pit and non-pit pixels (right). Pits shown in red were filtered from the candidate cache data set using probability and area thresholds determined from logistic regression and a pseudo accuracy assessment.
(JPG)

S2 Fig Form of the 5x5 kernel used in convolution of the resulting median-removed DTM which emphasized differences between the center pixels and their neighbors, accentuating low-lying areas. (JPG)

S3 Fig Lidar DTM and modified images for an approximately 12.5 hectare area on the east side of Douglas Lake. The lidar DTM (left) shows locations of cache pit clusters after automated and statistical filtering. Clusters shown in red were manually filtered based on visual assessment of each individual cluster, while those shown in green were included in the final count of cache pit clusters.
(JPG)

S1 Table Pseudo accuracy assessment of individual cache pit probability and area thresholds. For each area threshold, the probability threshold with the highest accuracy is reported. Thresholds of area 13 and probability 0.58 were selected (in **bold**) for high accuracy as well as high proportion of pits (positives) to non-pits (false positives).
(PDF)

S2 Table Pseudo accuracy assessment of cache pit clusters probability thresholds. A probability threshold of 0.51 (in **bold**) was selected because of high accuracy, high proportion of positively identified clusters to false positive clusters, and few omission errors.
(PDF)

S3 Table Comparison of Compound Topographic Index (CTI) values of pixels containing cache pits and pixels underlying randomly selected coordinate pairs. (PDF)
