# Supplementary figures and images for: Detecting Precontact Anthropogenic Microtopographic Features in a Forested Landscape with Lidar: A Case Study from the Upper Great Lakes Region, AD 1000-1600
Source: PLoS One. 2016 Sep 1;11(9):e0162062. doi: 10.1371/journal.pone.0162062 (PMC5008683; doi:10.1371/journal.pone.0162062)

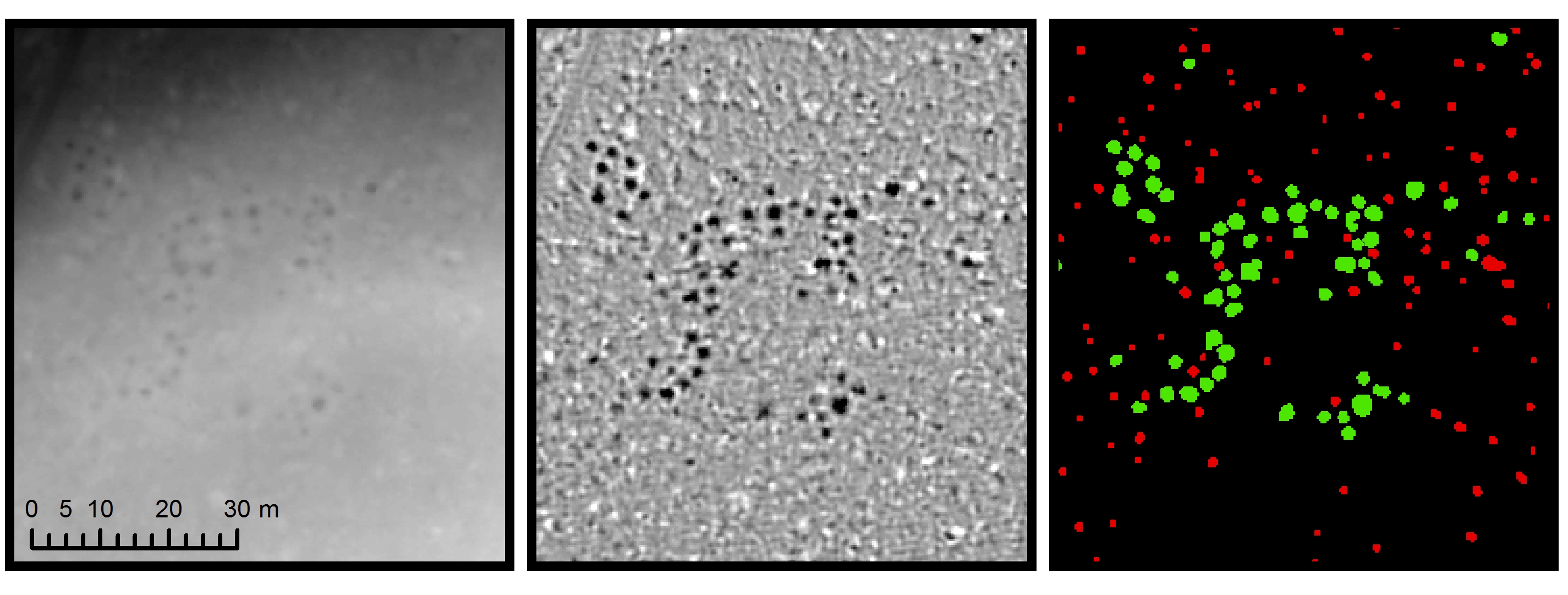

Supplement: S1 Fig — The lidar DTM (left) shows slight depressions, accentuated in DTMmod (middle), which was used to generate a binary image of pit and non-pit pixels (right). Pits shown in red were filtered from the candidate cache data set using probability and area thresholds determined from logistic regression and a pseudo accuracy assessment. (JPG) [file pone.0162062.s001.jpg]

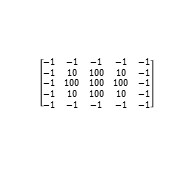

Supplement: S2 Fig — (JPG) [file pone.0162062.s002.JPG]

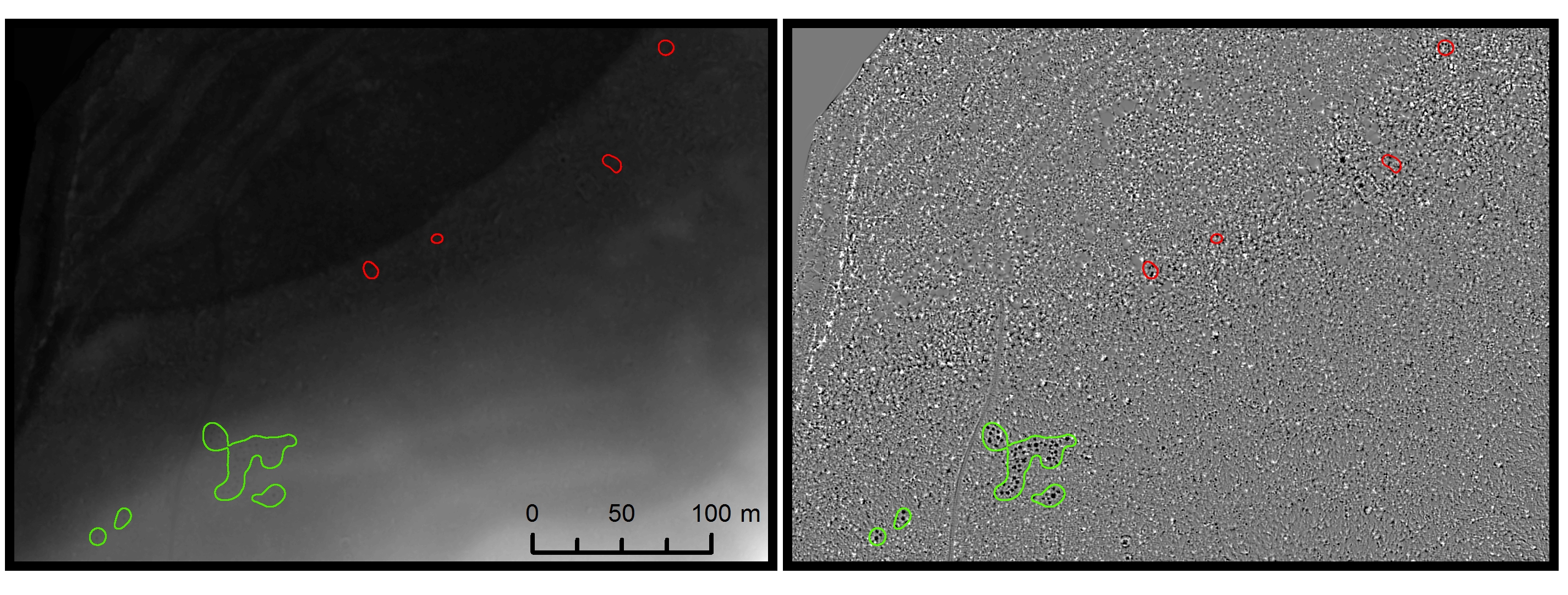

Supplement: S3 Fig — The lidar DTM (left) shows locations of cache pit clusters after automated and statistical filtering. Clusters shown in red were manually filtered based on visual assessment of each individual cluster, while those shown in green were included in the final count of cache pit clusters. (JPG) [file pone.0162062.s003.jpg]
