# Supplementary material for: Detecting Precontact Anthropogenic Microtopographic Features in a Forested Landscape with Lidar: A Case Study from the Upper Great Lakes Region, AD 1000-1600
Source: PLoS One. 2016 Sep 1;11(9):e0162062. doi: 10.1371/journal.pone.0162062 (PMC5008683; doi:10.1371/journal.pone.0162062)
Supplement: S1 Table — For each area threshold, the probability threshold with the highest accuracy is reported. Thresholds of area 13 and probability 0.58 were selected (in bold) for high accuracy as well as high proportion of pits (positives) to non-pits (false positives). (PDF) [file pone.0162062.s004.pdf]

a	P	Pits Count	Non-pits Count	Omission	Commission	A	Proportion
10	0.36	308	81	53	81	0.697	0.792
10	0.37	308	81	53	81	0.697	0.792
10	0.38	308	81	53	81	0.697	0.792
11	0.39	297	71	64	71	0.688	0.807
12	0.39	290	67	71	67	0.678	0.812
**13**	**0.58**	**261**	**32**	**100**	**32**	**0.664**	**0.891**
14	0.58	250	29	111	29	0.641	0.896
15	0.58	243	25	118	25	0.630	0.907
16	0.58	228	22	133	22	0.595	0.912
17	0.58	219	19	142	19	0.576	0.920
18	0.59	205	17	156	17	0.542	0.923
19	0.59	197	13	164	13	0.527	0.938

a, area; **P**, probability; **A**, accuracy
