# Supplementary material for: Detecting Precontact Anthropogenic Microtopographic Features in a Forested Landscape with Lidar: A Case Study from the Upper Great Lakes Region, AD 1000-1600
Source: PLoS One. 2016 Sep 1;11(9):e0162062. doi: 10.1371/journal.pone.0162062 (PMC5008683; doi:10.1371/journal.pone.0162062)
Supplement: S2 Table — A probability threshold of 0.51 (in bold) was selected because of high accuracy, high proportion of positively identified clusters to false positive clusters, and few omission errors. (PDF) [file pone.0162062.s005.pdf]

P	Pits Count	Non-pits Count	Omission	Commission	A	Proportion
0	50	59	0	59	0.459	0.459
0.01	50	26	0	26	0.658	0.658
0.02	49	25	1	25	0.653	0.662
0.03	49	25	1	25	0.653	0.662
0.04	49	23	1	23	0.671	0.681
0.05	49	23	1	23	0.671	0.681
0.06	49	22	1	22	0.681	0.690
0.07	49	22	1	22	0.681	0.690
0.08	49	21	1	21	0.690	0.700
0.09	49	19	1	19	0.710	0.721
0.1	49	18	1	18	0.721	0.731
0.11	49	18	1	18	0.721	0.731
0.12	49	18	1	18	0.721	0.731
0.13	49	18	1	18	0.721	0.731
0.14	49	17	1	17	0.731	0.742
0.15	49	17	1	17	0.731	0.742
0.16	49	16	1	16	0.742	0.754
0.17	49	16	1	16	0.742	0.754
0.18	49	16	1	16	0.742	0.754
0.19	49	16	1	16	0.742	0.754
0.2	49	16	1	16	0.742	0.754
0.21	49	14	1	14	0.766	0.778
0.22	49	13	1	13	0.778	0.790
0.23	49	13	1	13	0.778	0.790
0.24	49	13	1	13	0.778	0.790
0.25	49	13	1	13	0.778	0.790
0.26	49	13	1	13	0.778	0.790
0.27	49	13	1	13	0.778	0.790
0.28	49	13	1	13	0.778	0.790
0.29	49	13	1	13	0.778	0.790
0.3	49	13	1	13	0.778	0.790
0.31	49	12	1	12	0.790	0.803
0.32	49	12	1	12	0.790	0.803
0.33	49	11	1	11	0.803	0.817
0.34	49	10	1	10	0.817	0.831
0.35	49	10	1	10	0.817	0.831
0.36	49	10	1	10	0.817	0.831
0.37	49	9	1	9	0.831	0.845
0.38	48	9	2	9	0.814	0.842
0.39	48	9	2	9	0.814	0.842

0.4	48	9	2	9	0.814	0.842
0.41	48	9	2	9	0.814	0.842
0.42	48	9	2	9	0.814	0.842
0.43	48	9	2	9	0.814	0.842
0.44	48	9	2	9	0.814	0.842
0.45	48	9	2	9	0.814	0.842
0.46	48	9	2	9	0.814	0.842
0.47	48	8	2	8	0.828	0.857
0.48	47	8	3	8	0.810	0.855
0.49	47	7	3	7	0.825	0.870
0.5	47	7	3	7	0.825	0.870
**0.51**	**47**	**7**	**3**	**7**	**0.825**	**0.870**
0.52	46	7	4	7	0.807	0.868
0.53	45	6	5	6	0.804	0.882
0.54	44	6	6	6	0.786	0.880
0.55	43	6	7	6	0.768	0.878
0.56	41	6	9	6	0.732	0.872
0.57	41	6	9	6	0.732	0.872
0.58	41	6	9	6	0.732	0.872
0.59	41	6	9	6	0.732	0.872
0.6	40	5	10	5	0.727	0.889
0.61	40	5	10	5	0.727	0.889
0.62	40	5	10	5	0.727	0.889
0.63	40	5	10	5	0.727	0.889
0.64	39	5	11	5	0.709	0.886
0.65	38	5	12	5	0.691	0.884
0.66	38	3	12	3	0.717	0.927
0.67	38	3	12	3	0.717	0.927
0.68	38	3	12	3	0.717	0.927
0.69	37	3	13	3	0.698	0.925
0.7	37	2	13	2	0.712	0.949
0.71	37	2	13	2	0.712	0.949
0.72	37	2	13	2	0.712	0.949
0.73	37	2	13	2	0.712	0.949
0.74	37	2	13	2	0.712	0.949
0.75	37	2	13	2	0.712	0.949
0.76	37	2	13	2	0.712	0.949
0.77	36	2	14	2	0.692	0.947
0.78	36	2	14	2	0.692	0.947
0.79	36	2	14	2	0.692	0.947
0.8	36	2	14	2	0.692	0.947

0.81	34	2	16	2	0.654	0.944
0.82	34	2	16	2	0.654	0.944
0.83	33	2	17	2	0.635	0.943
0.84	33	2	17	2	0.635	0.943
0.85	33	2	17	2	0.635	0.943
0.86	33	2	17	2	0.635	0.943
0.87	33	2	17	2	0.635	0.943
0.88	32	1	18	1	0.627	0.970
0.89	31	1	19	1	0.608	0.969
0.9	31	1	19	1	0.608	0.969
0.91	30	1	20	1	0.588	0.968
0.92	28	1	22	1	0.549	0.966
0.93	26	0	24	0	0.520	1.000
0.94	21	0	29	0	0.420	1.000
0.95	20	0	30	0	0.400	1.000
0.96	16	0	34	0	0.320	1.000
0.97	14	0	36	0	0.280	1.000
0.98	10	0	40	0	0.200	1.000
0.99	5	0	45	0	0.100	1.000

P, probability; **A**, accuracy
