# Supplementary material for: Detecting Precontact Anthropogenic Microtopographic Features in a Forested Landscape with Lidar: A Case Study from the Upper Great Lakes Region, AD 1000-1600
Source: PLoS One. 2016 Sep 1;11(9):e0162062. doi: 10.1371/journal.pone.0162062 (PMC5008683; doi:10.1371/journal.pone.0162062)
Supplement: S3 Table — (PDF) [file pone.0162062.s006.pdf]

Pixel Size	Cache/Random	N	Min	Median	Mean	Max	D	p-value
10 m	Cache	185	4.849	7.575	7.864	13.34	0.2902	<0.001
	Random	349	5.042	8.637	8.966	16.2		
30 m	Cache	85	5.199	7.439	7.515	11.03	0.3816	<0.001
	Random	349	4.93	8.761	9.042	15.46		
90 m	Cache	49	5.595	7.446	7.53	10.81	0.5034	<0.001
	Random	348	5.528	8.953	9.202	15.24		
